# A Novel CACNA1A Nonsense Variant [c.4054C>T (p.Arg1352^⁎^)] Causing Episodic Ataxia Type 2

**DOI:** 10.1155/2018/5802650

**Published:** 2018-03-11

**Authors:** Sean Lance, Stuart Mossman, Gemma Poke

**Affiliations:** Wellington Hospital, Wellington, New Zealand

## Abstract

Episodic ataxia is a heterogenous group of uncommon neurological disorders characterised by recurrent episodes of vertigo, dysarthria, and ataxia for which a variety of different genetic variations have been implicated. Episodic ataxia type two (EA2) is the most common and also has the largest number of identified causative genetic variants. Treatment with acetazolamide is effective in improving symptoms, so accurate diagnosis is essential. However, a large proportion of patients with EA2 have negative genetic testing. We present a patient with a typical history of EA2 who had a novel variant in the CACNA1A gene not previously described. Report of such variations is important in learning more about the disease and improving diagnostic yield for the patient.

## 1. Case Report

We describe the case of a 47-year-old male who presented to neurology clinic with recurrent episodes of acute vertigo and unsteadiness, associated with severe nausea and vomiting. The symptoms of vertigo and ataxia typically lasted 1–3 hours, with persistent nausea often for the following 24 hours. He was having several episodes per week, with the observation by himself that they were more likely to occur in warmer weather and on exertion, with an improvement in symptoms when attempting to cool himself.

These episodes had been recurrent since approximately age 10 and were previously labelled as seizures. However, there was no reported loss of consciousness or observed seizure-like activity. Additionally, treatment with an unknown anticonvulsant (presumed to be phenytoin given his age) in his teenage years had actually exacerbated the symptoms.

Other relevant history includes intermittent migraines without any other associated neurological symptoms. He had a mild intellectual disability recognised since childhood which was unquantified. The patient was unaware of any family members being affected by similar problems and specifically no reports of suffering from ataxia, seizures, or migraine. No further history was obtainable directly from his family members.

On examination there was mild dysarthria. Eye movements revealed slightly jerky pursuit with a small target which was normal with a larger target; saccades were normal and there was no nystagmus. Head impulse test was normal. He had mild intention tremor on finger nose testing and gait assessment revealed a broad based ataxic gait with an inability to tandem walk with an otherwise normal neurological examination.

MRI brain was unremarkable. Previous EEG from 1991 was normal.

Given his history of recurrent attacks of vertigo and ataxia, he was started on acetazolamide with a significant reduction in symptoms, in both severity and frequency, to once per month, compared to several times per week.

Genetic testing showed a novel heterozygous variant in exon 25 of the* CACNA1A* gene, c.4054C>T (p.Arg1352^*∗*^). Nonsense mutations are an established cause of episodic ataxia type 2, and the variant did not appear in the GnomAD population database. This variant was therefore predicted to be pathogenic. This finding confirmed the diagnosis of episodic ataxia type 2 and represents another newly identified variant which can now be searched for in other patients.

Referral to the genetic service was made, with his family members declining screening.

## 2. Discussion

Episodic ataxia (EA) is rare, with incidence thought to be less than 1 per 100,000 [1]. Several different types of episodic ataxia have been described, with types one and two making up the majority of cases. EA type two (EA2) is the most common type and characterised by recurrent episodes of vertigo, ataxia, and dysarthria which typically last for a period of hours (as compared to EA type one (EA1) where symptoms last only minutes) [1, 2]. Additionally, findings of ataxia, dysarthria, and nystagmus may persist in between attacks in EA2 but are not classically a persistent feature of EA1. Attacks may be precipitated by various environmental factors including heat and exertion, as well as drugs such as caffeine and phenytoin [1, 2].

The underlying pathological mechanism for the episodic ataxias likely relates to abnormal neurotransmission, explained by the seven described subtypes of EA having underlying variants in genes coding for various channelopathies, pumps, and transporters [1].

In EA2, the genetic abnormality involves the* CACNA1A* gene located on chromosome 19p13. This gene encodes the alpha-1A subunit of the P/Q-type voltage-gated calcium channel. This is found throughout the nervous system but in higher density in the cerebellum, accounting for the prominent cerebellar symptoms and signs [3].

There have been over 50 different mutations described with the majority being nonsense mutations leading to loss of function of this channel [1–4]. These lead to reduced calcium currents and alteration in calcium dependent neurotransmitter release and subsequently the typical phenotypic presentation as described.

In addition to EA2, the* CACNA1A *gene is implicated in at least two other autosomal dominant neurological disorders: familial hemiplegic migraine type 1 (FHM1) and spinocerebellar ataxia type 6 (SCA6). In general, missense mutations are associated with FHM1 and trinucleotide (CAG) expansions associated with SCA6. However, a number of reports more recently have identified a significant lack of genotype-phenotype correlation, for example, missense mutations causing EA2 [5–7]. Also reported is the significant variability of clinical phenotype within families who have the same underlying mutation [8], a complex feature of the* CACNA1A *gene which is not yet fully understood.

Additionally,* CACNA1A *gene mutations are known to be associated with epilepsy and biallelic mutations have been reported with an epileptic encephalopathy associated with progressive neurological decline, adding further to the complexity of this gene and the wide phenotypic variability [9].

Despite multiple mutations being described, 30–50% of patients with the classic clinical presentation of EA2 have negative genetic testing [1–3]. This observation, as well as the lack of genotype-phenotype correlation, makes diagnosis and subsequent familial screening difficult.

Our patient had a typical clinical presentation with typical associated features such as migraine and interictal dysarthria and ataxia. Initially thought to represent seizures, the description of his events fit well with a diagnosis of EA2 and also worsening of symptoms in EA2 is seen with phenytoin (which we suspect he was treated with when he was younger, although we were not able to confirm). Additionally, intellectual disability and psychological symptoms are often seen in EA. These factors and the predicted response to acetazolamide make the diagnosis of EA2 reasonably secure. The nonsense variant in* CACNA1A*, whilst previously undescribed, is considered to be pathogenic.

With a large number of patients with EA2 not having an identifiable genetic abnormality a number of whole-genome sequencing studies are attempting to identify novel mutations in such patients. Maksemous et al. identified nine novel variants in 31 patients with EA2, of which six were missense changes [10]. Although it is unclear whether all of these new variants were likely to be pathogenic, these results do highlight the high proportion of patients* without *a known genetic mutation (52%) and also that variants in genes other than* CACNA1A* may well contribute to the EA2 phenotype [10].

## 3. Conclusion

The identification of a* CACNA1A *variant such as ours is important in the further characterisation of this disorder and can offer another target for testing in similar patients and those in whom the genetic abnormality remains unknown. This can assist with their ongoing management and with genetic counselling. Reporting of such variants and further research into whole-genome sequencing will help to expand our knowledge of EA2 and assist with genetic diagnosis and subsequent management.
